# Evaluating the impact of rock climbing on mental health and emotional well-being in adolescents

**DOI:** 10.3389/fpsyg.2024.1426654

**Published:** 2024-11-08

**Authors:** Hüseyin Gürer, Faruk Akçınar, Semiha Cömertoğlu Arslan, Serpil Akçınar, Mehmet Güllü, Özgür Eken, Ahmet Kurtoğlu, Mehmet Ilkım, Madawi H. Alotaibi, Safaa M. Elkholi

**Affiliations:** ^1^Department of Physical Education and Sports, Faculty of Sport Science, Inonu University, Malatya, Türkiye; ^2^Department of Coaching Education, Faculty of Sports Sciences, Inonu University, Malatya, Türkiye; ^3^Department of Child and Adolescent Psychiatry, Faculty of Medicine, Sutcu Imam University, Kahramanmaraş, Türkiye; ^4^Department of Physical Education and Sport Teaching, Faculty of Sports Sciences, Inonu University, Malatya, Türkiye; ^5^Department of Coaching Education, Faculty of Sport Science, Bandirma Onyedi Eylul University, Balikesir, Türkiye; ^6^Department of Rehabilitation Sciences, College of Health and Rehabilitation Sciences, Princess Nourah bint Abdulrahman University, Riyadh, Saudi Arabia

**Keywords:** rock climbing, mental health, anxiety, physical activity, emotional and behavioral problems

## Abstract

**Background:**

Rock climbing (RC) has gained attention as a therapeutic tool in psychiatric settings that merges physical exertion with mental engagement. It has potential to enhance mental health, through improved self-efficacy and social interaction, making it a novel intervention for addressing anxiety, depression, and behavioral issues in adolescents. This study aimed to investigate the effects of RC as a physical activity on anxiety, depression, and emotional and behavioral problems in adolescents.

**Methods:**

The current study included 57 athletes aged 14.5 ± 1.7 years and 91 adolescents aged 13.6 ± 1.2 years, matched for age and gender, who were not professionally involved in sport. In addition to the socio-demographic form, a detailed psychiatric assessment was carried out by the child psychiatrist; using the Schedule for Affective Disorders and Schizophrenia for School-Age Children Present and Lifetime Version (K-SADS-PL) to detect psychiatric conditions. The Revised Child Anxiety and Depression Scale-Child Version (RCADS-CV) and the Strengths and Difficulties Questionnaire (SDQ) were also administered to the adolescents in the study.

**Results:**

In the comparative analysis of the RCADS-CV outcomes between the athlete and control groups, the athletes demonstrated notably lower scores for both Separation Anxiety Disorder (SAD) and Generalized Anxiety Disorder (GAD), yielding *p*-values of < 0.001 and 0.031, respectively. Although the mean scores for social phobia, OCD, panic disorder, and MDD were lower in the athlete group, the differences were not statistically significant (*p* > 0.05). In the correlation analysis, a moderately significant correlation was found between the duration of doing sport and the scale scores for SAD (*p*:0.010), OCD (*p*:0.014), and panic disorder (*p*:0.016). There was no significant difference between groups in terms of SDQ scores.

**Conclusion:**

These results suggest that RC, through its unique combination of physical exertion and mental focus, may offer protective benefits against certain anxiety disorders among adolescents. Further studies should be conducted to explore the potential use of RC as a preventive program for both healthy adolescents, as well as those with psychiatric disorder.

## 1 Introduction

Physical activity is widely recognized for its positive impact on mental health (Mahindru et al., 2023), including the reduction of symptoms related to anxiety (Singh et al., 2023), depression (Gianfredi et al., 2020), and emotional distress (Dai et al., 2021). Among the various forms of physical activity, rock climbing stands out as a particularly beneficial intervention for mental health due to its combination of physical exertion and mental engagement (MacKenzie et al., 2020; Wheatley, 2023). The sport not only requires focus and problem-solving but also fosters a sense of accomplishment, making it well-suited for addressing mental health challenges such as anxiety and depression.

Many adult mental disorders originate from childhood, making early interventions crucial for long-term mental health. Interventions, measures, and practices designed to enhance children’s coping skills can positively impact their mental health and provide significant benefits (Andermo et al., 2020).

Engaging in physical activity (PA) has been shown to have positive effects on the mental health of adolescents (Belcher et al., 2021). Meta-analyses of studies conducted in children and adolescents have consistently shown that engaging in PA is associated with lower levels of psychological distress, including depression, anxiety, stress, negative impact, and total psychological distress. PA has also been linked to higher levels of psychological well-being, including self-image, satisfaction with life, happiness, and overall psychological well-being (Hale et al., 2021; Vaquero-Solís et al., 2021; Granero-Jiménez et al., 2022; Merino et al., 2024). Conversely, increased sedentary behavior has been linked to higher levels of psychopathology and lower self-esteem in children and adolescents (Ibrahim et al., 2022; Medeiros et al., 2024). Regular participation in sport activities during adolescence has been associated with reduction in depression levels in adulthood, indicating that the positive effects of PA extend beyond the period during which it is performed (Miller et al., 2024). Research has shown that team sports, particularly those without esthetic purposes, are strongly associated with better mental health among young people (Rodriguez-Ayllon et al., 2019).

Although physical exercise has been recommended among the alternative treatment modalities for many mental health problems and the number of studies on this subject has been increased in recent years, there is no clear information about the differences in the level of effectiveness of different exercise modalities and sport branches (Kramer, 2020).

The interconnection between physical activity and mental health has been a subject of substantial interest within the scientific community, particularly in how various forms of exercise can affect psychological well-being (Belcher et al., 2021; Granero-Jiménez et al., 2022). Among the myriad of physical activities, rock climbing (RC) has emerged as an intriguing area of study, especially in its application within therapeutic studies (Karg et al., 2020; Draper et al., 2021). RC also known as bouldering, which is a sub-type of climbing sport, stands out as a relatively more researched subject. Currently, climbing sport has attracted more attention in clinical practice since it is often used as part of a general treatment plan for several health problems (Dorscht et al., 2019; Frühauf et al., 2021; Gassner et al., 2023). In addition to being one of the basic forms of movement of human nature, Rock climbing (RC) is a sport activity with both physical and psychological needs and consists of many sub-branches. RC is the most popular subtype of the fixed anchors placed at certain intervals on the climbing wall (Walker et al., 2020; Langer et al., 2023). RC, characterized by its demands for both physical endurance and mental concentration, offers a unique blend of challenges and rewards (Liu et al., 2022; Wheatley, 2023). Not only does it require significant physical strength and flexibility, but it also necessitates acute mental focus and problem-solving abilities, making it a comprehensive activity that engages the whole person (Heilmann, 2021; Kawashima et al., 2024). The potential of rock climbing to serve as a therapeutic modality stems from its ability to improve self-efficacy, enhance social interactions, and provide individuals with a sense of achievement and mastery over their environment (Houge Mackenzie and Brymer, 2020; Vreuls et al., 2022). Studies suggest that RC is associated with decreased depression (Rosołowska-Żak et al., 2024) and anxiety (Singh et al., 2023; Wheatley, 2023).

This study aimed to evaluate the anxiety and depression levels and emotional and behavioral problems in children and adolescents who engage in professional RC sports, to assess the potential impact of RC on mental health in this population. By doing so, we hope to contribute to the discussion on RC as a potential adjunctive treatment and prevention for mental health issues affecting adolescents. It is hypothesized that adolescents who engage in professional RC sports will exhibit significantly lower levels of Separation Anxiety Disorder (SAD) and Generalized Anxiety Disorder (GAD), compared to a control group of adolescents not engaged in professional sports.

## 2 Methods

The study was designed as a cross-sectional investigation and received approval from the Inonu University Health Sciences Non-Interventional Clinical Research and Publication Ethics Committee (Approval No: 2020/743). All participants and their legal guardians provided written informed consent following a comprehensive verbal explanation of the study’s aims and procedures. The study population comprised 57 athletes aged 11–16 years, all of whom regularly participated in rock climbing and were registered with the Turkey Mountaineering Federation. The control group consisted of 91 adolescents matched with the athletes based on age and gender, none of whom engaged in professional sports. A purposive sampling technique was employed to ensure that the sample included individuals fitting the study criteria: adolescents within the specified age range (11–16 years) who were either professional rock climbers or not engaged in any professional sports. The sample size was determined based on previous literature, which indicated that a minimum of 50 participants per group would be required to achieve adequate statistical power for detecting differences in mental health outcomes. Therefore, 57 rock climbers and 91 non-athletes were included in the final analysis. Adolescents with chronic medical conditions (e.g., cardiovascular or respiratory diseases) or a history of regular medication use, which could affect psychological mood, were excluded. A socio-demographic form, which captured details on sports history, weekly training volume, family characteristics, and medical history, was completed for each participant. A thorough psychiatric assessment was conducted by a pediatric psychiatrist using the Turkish version of the Schedule for Affective Disorders and Schizophrenia for School-Age Children-Present and Lifetime Version (K-SADS-PL). Additionally, all participants completed the Revised Child Anxiety and Depression Scale-Child Version (RCADS-CV) and the Strengths and Difficulties Questionnaire (SDQ) to evaluate anxiety, depression, and other psychological factors. Inclusion criteria specified adolescents aged 11–16, with the athlete group actively engaged in rock climbing and registered with the federation, and the control group consisting of age- and gender-matched non-athletes. Exclusion criteria encompassed chronic medical conditions and a history of regular medication use, as well as incomplete psychiatric assessments or failure to complete the required psychological measures (K-SADS-PL, RCADS-CV, SDQ).

### 2.1 Screening instruments

#### 2.1.1 Psychiatric assessment

The Schedule for Affective Disorders and Schizophrenia for School-Age Children-Present and Lifetime Version (K-SADS-PL) is a semi-structured interview form developed by Kaufman et al. (1997) to detect past and present mental disorders in children and adolescents (Kaufman et al., 1997). It is implemented through one to one interviews with parents and the child. Gokler et al. (2004) conducted a validity and reliability study of the Turkish version. This is a semi-structured interview and does not use a Likert scale. Instead, it involves direct interviews between the psychiatrist and the participant (and their parent), where the interviewer records the presence or absence of psychiatric symptoms based on the responses.

#### 2.1.2 Anxiety and depression

The Revised Child Anxiety and Depression Scale-Child Version (RCADS-CV) is a self-report questionnaire developed to screen anxiety disorders and depression in children and adolescents based on the DSM-IV criteria (Chorpita et al., 2000). The RCADS-CV uses a Likert-type scale with 4 response options. It includes a form that is filled out by the parent and child. It consists of 6 subscales and 47 items evaluating generalized anxiety disorder (GAD) (6 items), Separation Anxiety Disorder (SAD) (7 items), social phobia (9 items), panic disorder (9 items), obsessive-compulsive disorder (OCD) (6 items), and major depressive disorder (MDD) (10 items). Each item is scored between 0 and 3. Gormez et al. (2017) performed the Turkish validity and reliability study on children and adolescents between 7 and 18 years of age.

#### 2.1.3 Behavioral problems

The Strengths and Difficulties Questionnaire (SDQ) was used to assess positive and negative behavioral characteristics. The SDQ uses a 3-point Likert scale Goodman et al. (1998), developed the SDQ and its validity and reliability in Turkish was confirmed by Güvenir et al. (2008). The scale includes a parent form and teacher form for ages 4–16 and an adolescent form for ages 11–16, which is filled out by the adolescents themselves. It consists of 25 questions that evaluate positive and negative behavior characteristics, grouped under five subheadings: conduct problems, hyperactivity/inattention, emotional problems, peer problems, and prosocial behaviors. High scores in the prosocial behavior subheading indicate the individual’s strengths in the social field, while high scores in the other four areas indicate the severity of the problem in the relevant subheadings.

### 2.2 Statistical analysis

The data analysis was conducted using SPSS 25.0 software. Quantitative data were represented as means and standard deviations, whereas qualitative data were presented in the form of numbers and percentages. The normality of the dataset was assessed using the Kolmogorov–Smirnov test. Given that the data did not adhere to a normal distribution, the Mann–Whitney U Test was employed for the analysis of quantitative independent variables. Independent qualitative variables were analyzed using the chi-square test. Correlations between variables were determined using Spearman’s correlation coefficients, with a *p*-value of < 0.05 being considered indicative of statistical significance. For the specific case of OCD and SP, the assumption of homogeneity of variances, a prerequisite for the independent samples *t*-test, was evaluated. The Levene’s test indicated that variances between the distributions of OCD scores in the control and experimental groups were equal (*F* = 0.041, *p* > 0.05), thus validating the use of the independent samples *t*-test as detailed in the table below. It was observed that the scores for both the total scale and its subscales were normally distributed, justifying the application of the independent samples *t*-test. The assumption of homogeneity of variances for the independent samples *t*-test was again assessed. According to the Levene’s test results; the variances in the distributions of scores between the control and experimental groups were found to be equal across various dimensions (F SDQ_emotional = 2.502, *p* > 0.05; F SDQ_behavioral = 0.448, p > 0.05; F SDQ_attention = 0.178, *p* > 0.05; F SDQ_peer = 0.879, *p* > 0.05; F SDQ_social = 2.502, *p* > 0.05; F SDQ_total = 2.502, *p* > 0.05). The normality of distributions was initially examined, and as indicated above, while some distributions were found to follow a normal pattern, others did not. Consequently, analysis were conducted using Spearman’s correlation due to the mixed distribution patterns observed.

## 3 Results

The mean age of the athletes participating in the study was 14.5 ± 1.7 years, and the control group was 13.6 ± 1.2 years. 60% of the athletes and 49% of the control group were male. There was no statistically significant difference between the groups in terms of age and gender (*p* > 0.05). The athletes engaged in regular exercise on 3.7 ± 1.6 (min-max: 1–7) days per week and had been involved in various sports, including climbing, for an average of 3.8 ± 2.5 (min-max: 0.5–8) years. None of the adolescents in the control group participated in sports regularly. Detailed socio-demographic characteristics of the adolescents included in the study are shown in Table 1.

**Table 1:** Sociodemographic characteristics of athletes and control groups.

Sociodemographic variable	Athletes	Control group
Age (years), mean ± SD (min–max)	14.5 ± 1.7 (11–16)	13.6 ± 1.2 (11–16)
	***n* (%)**	***n* (%)**
**Gender**		
Female	23 (40)	47 (51)
Male	34 (60)	44 (49)
**Mother’s education level**		
Primary school	25 (44)	50 (55)
High school	10 (17)	10 (11)
University	22 (39)	31 (34)
**Father’s education level**		
Primary school	20 (35)	33 (37)
High school	7 (12)	25 (26)
University	30 (53)	33 (37)
**Income level**		
Low	5 (9)	20 (22)
Medium	23 (40)	35 (38)
High	29 (51)	36 (40)

### 3.1 Comparison of K-SADS-PL results

The comparison of K-SADS-PL results between athletes and controls showed that 41 athletes (71.9%) had no psychiatric symptoms. One athlete showed symptoms of specific phobia, one athlete showed symptoms of social phobia, and three athletes showed symptoms of GAD. In 10 athletes, symptoms indicated a possible ADHD diagnosis, but this could not be confirmed due to limitations in the study design. In the control group, two out of 91 children/adolescents exhibited symptoms consistent with major depressive disorder. Additionally, seven had a GAD, two had a specific phobia, and two had social phobia. According to the self-reports of the adolescents, eight of them met the diagnostic criteria for ADHD. However, this could not be confirmed with family and teacher reports due to the study design. There was no statistically significant difference found between athletes and controls in terms of K-SADS-PL results (*p* > 0.05). Table 2 presents a detailed comparison of the K-SADS-PL results between athletes and control groups.

**Table 2:** Comparison of K-SADS-PL results of athletes and control groups.

Diagnosis		Athletes *n* (%)	Control group *n* (%)	*p* value*
No diagnosis		41 (71.9)	70 (76.9)	0.741
With diagnosis	Attention deficit/hyperactivity disorder	10 (17.5)	8 (8.8)	
	Generalized anxiety disorder	3 (5.3)	7 (7.7)	
	Social phobia	1 (1.8)	2 (2.2)	
	Specific phobia	1 (1.8)	2 (2.2)	
	Major depressive disorder	1 (1.8)	2 (2.2)	
	Total	57 (100)	91 (100)	

*The chi-square test.

### 3.2 Evaluation of the RCADS-CV and SDQ scores

The comparison of RCADS-CV scores between athletes and the control group revealed that athletes had significantly lower scores for SAD and GAD (*p* < 0.001, *p*:0.031, respectively). However, there was no significant difference in scores for SP, OCD, PD, and MDD between the two groups (*p* > 0.05 for all scores).

The comparison of SDQ scores between athletes and the control group revealed that emotional problems, hyperactivity/inattention, peer problems, and total scores were higher, and prosocial behavior was lower in controls than in athletes. However, the difference was not statistically significant (*p* > 0.05).

Table 3 presents detailed comparison of RCADS-CV and SDQ scores between athletes and control groups.

**Table 3:** Comparison of RCADS-CV and SDQ scores between athletes and control groups.

	Athletes Mean ± SD	Control group Mean ± SD	*p* value
**RCADS-CV scores**			
Separation anxiety disorder	2.0	5.0	**<0.001** ^*^
Social phobia	7.11 ± 4.6	8.83 ± 5.9	0.060^**^
Obsessive-compulsive disorder	5.46 ± 4.3	6.22 ± 4.1	0.289^**^
Panic disorder	4.0	4.0	0.803^*^
Generalized anxiety disorder	5.0	6.0	**0.031** ^*^
Major depressive disorder	5.0	5.0	0.277^*^
**SDQ scores**			
Emotional problems	2.09 ± 2.0	2.73 ± 2.2	0.085^**^
Conduct problems	2.89 ± 1.8	2.40 ± 1.9	0.128^**^
Hyperactivity/inattention	3.49 ± 2.1	3.94 ± 2.2	0.225^**^
Peer problems	3.18 ± 2.1	3.40 ± 1.9	0.518^**^
Prosocial behavior	7.35 ± 2.2	7.14 ± 2.7	0.618^**^
Total score	19.02 ± 5.7	19.68 ± 4.9	0.469^**^

RCADS-CV: Revised Child Anxiety and Depression Scale-Child Version.

Bold font indicates statistical significance: *p* < 0.05.

### 3.3 Evaluation of the relationship between the duration of doing sports and emotional and behavioral problems in athletes

In the correlation analysis performed to evaluate the effect of the duration of regular sports (in years), including climbing sports, on emotional and behavioral problems; there was a moderately significant negative correlation between the duration of doing sports and RCADS-CV SAD, OCD, and PD scores (*p*:0.010, *p*:0.014, *p*:0.016, respectively). However, there was no significant correlation between other RCADS-CV subscales, SDQ scores, and the duration of doing sports (*p* > 0.05). Table 4 presents the data regarding the correlation analysis.

**Table 4:** Evaluation of the relationship between regular sports duration and scale scores in athletes.

RCADS-CV scores	The athletes engaged in sports	
	*r* ^a^	*p* value^a^
Separation anxiety disorder	−0.431**	**0.010**
Social phobia	−0.292	0.089
Obsessive-compulsive disorder	−0.413*	**0.014**
Panic disorder	**−0.403** ^*^	**0.016**
Generalized anxiety disorder	−0.068	0.698
Major depressive disorder	−0.293	0.087
**SDQ scores**		
Emotional problems	−0.179	0.305
Conduct problems	−0.249	0.150
Hyperactivity/inattention	−0.058	0.742
Peer problems	−0.215	0.214
Prosocial behavior	0.009	0.957
Total score	−0.253	0.143

**Correlation is significant at the 0.01 level, * Correlation is significant at the 0.05 level, a: Spearman correlation analysis, SDQ: Strengths and Difficulties Questionnaire, RCADS-CV: Revised Child Anxiety and Depression Scale-Child Version, r: Spearman’s rank correlation coefficient, Bold font indicates statistical significance.

### 3.4 Evaluation of the social phobia and OCD scores

While the differences in scores for social phobia and OCD were not statistically significant, the trend of lower scores in the athlete group suggests that rock climbing may still have some positive influence on these conditions. Non-significant results do not negate the possibility of a meaningful effect, particularly in real-world settings where multiple factors influence mental health outcomes. Engaging in a mentally challenging activity like rock climbing could improve cognitive flexibility and stress resilience, which are factors known to help in managing OCD and social anxiety. Although this study did not demonstrate significant statistical differences, it provides a foundation for future investigations into the mechanisms behind these trends.

## 4 Discussion

To our knowledge, this study is the first study to evaluate the effects of RC on anxiety and depression levels, as well as emotional and behavioral problems, in adolescents. The study found no significant difference in the diagnosis of psychiatric disorders between the groups. However, the study group had significantly lower scores for SAD and GAD on the RCADS-CV scale compared to the control group. Furthermore, although not statistically significant, the RC group exhibited lower scores for anxiety-related disorders, including social phobia, OCD, panic disorder, and MDD. Additionally, a significant correlation was found between the duration of doing sports and SAD, OCD, and panic disorder. As the duration of regular exercise increased, there was a significant improvement in the scale scores of these disorders. These findings are consistent with previous literature indicating that PA can have a positive impact on anxiety disorders in adolescents. Additionally, although not statistically significant, the results suggest that participation in sports may have a positive effect on the psychological well-being of children, as athletes had better scores for hyperactivity/inattention, emotional problems, peer problems, and prosocial behaviors.

In addition to the cognitive behavioral therapy (CBT) and antidepressants recommended in the guidelines for depressive disorder, PA is one of the leading alternative interventions that support complete recovery either alone or as an adjunctive treatment strategy (Yokoyama et al., 2023). Meta-analysis conducted on adolescents have reported that PA has minor and moderate effects on mental illnesses (Wang et al., 2022; Yang et al., 2022; Zhang et al., 2022). Furthermore, a meta-analysis investigating the potential protective effect of PA on depression in adolescents found that a higher level of PA was associated with fewer depressive symptoms. Previous studies have reported that PA is linked to a reduction in depression symptoms and has weak but significant protective effects on future depressive symptoms, particularly when performed regularly during childhood and adolescence (Wang et al., 2022). In our study, the depression scores on the RCADS-CV scale were lower in the athletes, but the difference was not statistically significant.

Several meta-analysis studies have reported a consistent inverse relationship between PA and anxiety, and PA is associated with a small to moderate decrease in anxiety levels (Pearce et al., 2022; Zhang et al., 2022; Singh et al., 2023). Furthermore, sedentary behavior has been linked to anxiety disorders, as demonstrated by Teychenne et al. (2015). It is also known that PA can reduce high levels of anxiety sensitivity, increase physiological resilience to stress, improve sleep, and positively affect the sense of mastery and self-efficacy (Carter et al., 2021). In a study evaluating 11,110 adolescents, McMahon et al. (2017) showed that there is a negative correlation between PA and both depressive symptoms and anxiety and a positive correlation between well-being. Research has shown that regular PA can improve mental health by reducing symptoms of depression and anxiety in both boys and girls (McMahon et al., 2017). Our findings support the view that increases in PA can provide significant benefits to mental health.

Small sample studies and observations on RC used for therapeutic purposes in some psychiatric hospitals in Germany (Luttenberger et al., 2015) is reported to be associated with positive effects on anxiety (Dodd and Lester, 2021), ADHD (Vysniauske et al., 2020), depression (DelGrande et al., 2020), eating disorders (Melissa et al., 2020), cognition (Roberts et al., 2022), self-esteem (Wheatley, 2023), and in the social field (Gassner et al., 2023). Kleinstäuber et al. (2017) investigated the impact of a 2.5-h RC session on negative emotional states, positive emotional states, and coping feelings (such as pride and self-confidence) in 40 adults with seasonal emotional disorder (SED). The study found that RC had acute mood-regulating effects, with a significant increase in positive affect and coping emotions, and a significant decrease in negative affect and depression compared to the control group. In a randomized controlled study, Karg et al. (2020) compared the effects of RC intervention with a home-based physical exercise program in depressive disorder. Both physical activities caused a significant decrease in depressive symptoms during the 10-week intervention period. However, RC intervention was found to be more effective than physical exercise (Karg et al., 2020). In addition, during the 10-week intervention period, participants in the RC group showed significant positive changes in terms of anxiety, body image, active and passive coping, global self-esteem, and interpersonal sensitivity. These findings suggest that sports that require levels of high concentration and coordination and are associated with achieving a goal may have short-term emotional regulatory effects (Kleinstäuber et al., 2017). However, there is a lack of studies examining the effects of RC on mental disorders in children. Existing adult studies have primarily focused on depression and have typically been conducted with patient groups. Moreover, most studies have relied on scale evaluations rather than comprehensive psychiatric evaluations. Thus, the impact of RC on the mental health of healthy children and adolescents remains largely unexplored. Although there are few studies in the literature on the relationship between RC and anxiety in adults, no studies have been conducted on children to date. One study aimed to investigate the chronic effects of 8-week RC training on cognitive and physical anxiety and self-confidence levels in healthy sedentary adults. The results showed that RC significantly reduced cognitive and somatic anxiety and increased self-confidence. The authors suggested that the sense of self-efficacy developed through climbing may be a contributing factor in reducing cognitive and somatic anxiety (Ewert and Aras, 2016). Our study found that the SAD and GAD scores in the Anxiety-Depression Scale were significantly lower in the RC group compared to the control group, which is consistent with findings in the literature. Furthermore, the mean scores of social phobia, OCD, panic disorder, and other anxiety-related disorders were also lower in the RC group, although these differences were not statistically significant. Moreover, there was a moderately significant negative correlation between the duration of doing sports and the scores of SAD, OCD, and Panic Disorder.

Although RC is a physical sport, many views consider it a mental sport. In the studies, many opinions were presented as potential common mechanisms covering the psycho-social-biological factors defined by rock climbing. Many studies have shown that RC has positive effects including cognitive abilities (Whitaker et al., 2020), self-confidence, self-esteem, self-efficacy (MacKenzie et al., 2020; Vreuls et al., 2022), and social skills (Wheatley, 2023) which are known to be key structures that are thought to be effective in mental illnesses (Rosołowska-Żak et al., 2024). Climbing develops coordination abilities by forcing the entire human system (Liu et al., 2022). Exercises that require coordination are thought to have specific effects on cognitive abilities such as concentration (Whitaker et al., 2020). Unlike many other team sports, RC requires individuals to take risks and take full responsibility for their success or failure during RC activity. This situation provides individuals the opportunity to observe psychological effects as well as physiological limits and strengths (Ewert and Aras, 2016). It offers individuals an opportunity to learn more actively and to set realistic goals (Dorscht et al., 2019). It also supports the individual’s belief in himself and his abilities, the power to manage fear and anxiety, and solve current problems, awareness of existing boundaries, attention and focus, and improves social aspects such as trusting his partner, sense of responsibility and cooperation. It is thought that regular participation in RC will affect health positively (Hale et al., 2021; Mahindru et al., 2023). In a study investigating the relationship between vigorous PA and perceived stress, mental health, and socializing (evaluated using the number of close friends and hours spent for socialization) on 14,706 college students, strong PA was shown to have a positive effect on perceived stress and mental health. In addition, socialization was thought to mediate the relationships between vigorous PA and mental health and vigorous PA and perceived stress. Therefore, socialization stands out as an important factor in the relationship between PA and mental Health (VanKim and Nelson, 2013). In our study, parallel to the literature, more positive results were obtained in terms of attention deficit and excessive mobility, emotional problems, peer problems, and social behaviors, in the study group performing sports compared to the control group. Considering the positive effects of these factors on the mental health of adolescents, we suggest that RC is an important step in protecting the youth from mental disorders.

In summary, our study demonstrates that rock climbing (RC) can positively influence anxiety-related disorders, particularly separation anxiety disorder (SAD) and generalized anxiety disorder (GAD), in adolescents. While these findings align with existing literature on physical activity’s benefits for mental health, they also underscore the unique potential of RC due to its combination of physical and cognitive challenges. Future research should explore the long-term effects of RC, investigate its impact on other mental health conditions such as depression, and examine its application in broader adolescent populations, including those with varying psychiatric disorders. Expanding the sample size and including family-based assessments could further enhance our understanding of the psychosocial benefits of RC.

A key limitation of this study is the relatively small sample size, which may affect the generalizability of the findings. Additionally, the reliance on self-report measures such as the RCADS-CV and SDQ scales introduces the possibility of response biases, such as social desirability or recall bias, which could influence the accuracy of the data. Another significant limitation is the lack of longitudinal data. A longitudinal follow-up study would provide deeper insights into the long-term mental health benefits of rock climbing (RC) and help clarify whether the positive effects observed in this study are sustained over time.

## 5 Conclusion

In conclusion, this study provides compelling evidence that rock climbing (RC) can significantly reduce anxiety-related disorders, particularly separation anxiety disorder (SAD) and generalized anxiety disorder (GAD), in adolescents. These findings suggest that RC could be considered a valuable addition to sports-based interventions for mental health. Given the unique combination of physical and cognitive engagement that RC offers, it holds promise as a complementary therapy in clinical practice. Integrating RC into mental health interventions could provide an alternative or adjunctive treatment for adolescents struggling with anxiety, potentially enhancing traditional approaches like cognitive behavioral therapy (CBT). Future research and clinical applications should focus on expanding these interventions to more diverse populations and assessing their long-term benefits.

## Data Availability

The raw data supporting the conclusions of this article will be made available by the authors, without undue reservation.
